# Retraction: Causes of Cancer in the World: Comparative Risk Assessment of Nine Behavioral and Environmental Risk Factors

**DOI:** 10.7759/cureus.r139

**Published:** 2024-04-19

**Authors:** Khizer K Ansari, Asha Jha

**Affiliations:** 1 Pharmacology, Jawaharlal Nehru Medical College, Datta Meghe Institute of Medical Sciences, Wardha, IND

This article has been retracted by the Editor-in-Chief. The title and figures have been taken directly from a 2005 article published in Lancet: https://doi.org/10.1016/S0140-6736(05)67725-2. Additionally, the article appears to follow the Lancet article on a sentence-by-sentence basis with evidence of tortured phrases. The journal regrets that these issues were not identified during the plagiarism check at the time of submission. The authors agree with this decision to retract.

